# Comparative Molecular Docking, Molecular Dynamics and Adsorption–Release Analysis of Calcium Fructoborate and Alendronate Salts on Hydroxyapatite and Hydroxyapatite–Titanium Implants

**DOI:** 10.3390/biomedicines14010044

**Published:** 2025-12-24

**Authors:** Diana-Maria Trasca, Ion Dorin Pluta, Carmen Sirbulet, Renata Maria Varut, Cristina Elena Singer, Denisa Preoteasa, George Alin Stoica

**Affiliations:** 1 Department of Internal Medicine, University of Medicine and Pharmacy of Craiova, 200349 Craiova, Romania; 2 Faculty of Medical and Behavioral Sciences, Constantin Brâncuși University of Târgu Jiu, 210185 Târgu Jiu, Romania; 3 Discipline of Anatomy, Department of Anatomy, University of Medicine and Pharmacy of Craiova, 200349 Craiova, Romania; 4 Research Methodology Department, Faculty of Pharmacy, University of Medicine and Pharmacy of Craiova, 200349 Craiova, Romania; 5 Department of Mother and Baby, University of Medicine and Pharmacy of Craiova, 200349 Craiova, Romania; 6 Clinical Pharmacist, Vâlcea County Emergency Hospital, 240292 Râmnicu Vâlcea, Romania; 7 Department of Pediatric Surgery, Faculty of Medicine, University of Medicine and Pharmacy of Craiova, 200349 Craiova, Romania

**Keywords:** calcium fructoborate, alendronate salts, hydroxyapatite HAp–titanium, surface adsorption, drug release, molecular docking, molecular dynamics

## Abstract

**Background/Objectives:** Hydroxyapatite (HAp)-based implants and HAp–titanium (HApTi) composites are widely used in orthopedic and dental applications, but their long-term success is limited by peri-implant bone loss. Local delivery of osteoactive molecules from implant surfaces may enhance osseointegration and reduce periprosthetic osteolysis. This study combined in silico modeling and experimental assays to compare calcium fructoborate (CaFb), sodium alendronate, and calcium alendronate as functionalization agents for HAp and HApTi implants. **Methods:** Molecular docking (AutoDock 4.2.6) and 100 ns molecular dynamics (MD) simulations (AMBER14 force field, SPC water model) were performed to characterize ligand–substrate interactions and to calculate binding free energies (ΔG_binding) and root mean square deviation (RMSD) values for ligand–HAp/HApTi complexes. HAp and HApTi discs obtained by powder metallurgy were subsequently functionalized by surface adsorption with CaFb or alendronate salts. The amount of adsorbed ligand was determined gravimetrically, and in vitro release profiles were quantified by HPTLC–MS for CaFb and by HPLC after FMOC derivatization for alendronates. **Results:** CaFb–HAp and CaFb–HApTi complexes showed the lowest binding free energies (−1.31 and −1.63 kcal/mol, respectively), indicating spontaneous and stable interactions. For HAp-based complexes, the mean ligand RMSD values over 100 ns were 0.27 ± 0.17 nm for sodium alendronate, 0.72 ± 0.28 nm for calcium alendronate (range 0.35–1.10 nm), and 0.21 ± 0.19 nm for CaFb (range 0.15–0.40 nm). For HApTi-based complexes, the corresponding RMSD values were 0.30 ± 0.15 nm for sodium alendronate, 0.72 ± 0.38 nm for calcium alendronate and 0.26 ± 0.14 nm for CaFb. These distributions indicate that CaFb and sodium alendronate maintain relatively stable binding poses, whereas calcium alendronate shows larger conformational fluctuations, consistent with its less favorable binding energies. Experimentally, CaFb exhibited the greatest chemisorbed amount and percentage on both HAp and HApTi, followed by sodium and calcium alendronate. HApTi supported higher loadings than HAp for all ligands. Release studies demonstrated a pronounced burst and rapid plateau for both alendronate salts, whereas CaFb displayed a slower initial release followed by a prolonged, quasi-linear liberation over 14 days. **Conclusions:** The convergence between in silico and adsorption–release data highlights CaFb as the most promising candidate among the tested ligands for long-term functionalization of HAp and HApTi surfaces. Its stronger and more stable binding, higher loading capacity and more sustained release profile suggest that CaFb-coated HApTi implants may provide a favorable basis for future in vitro and in vivo studies aimed at improving osseointegration and mitigating periprosthetic osteolysis, although direct evidence for osteolysis prevention was not obtained in the present work.

## 1. Introduction

HAp-based implants are widely used in an increasing number of patients in orthopedic and dental medicine [1,2,3]. Reinforcement with particles, short fibers, and long fibers has been utilized to create HAp biocomposites with superior mechanical properties. Bio-inert Ti particles are useful for reinforcing HAp, having a positive effect on the mechanical and biological properties of the biocomposites [4,5,6].

The primary cause of deterioration in components of orthopedic prosthesis is periprosthetic osteolysis. Avoiding periprosthetic bone loss is achieved through therapeutic schemes that use antiresorptive drugs or newly discovered drugs for bone health. Bisphosphonates are a class of antiresorptive drugs known to inhibit osteoclastic activity. A series of preclinical studies have shown a substantial increase in mineral bone density in the periprosthetic area after the use of high drug doses, comparable to those administered to patients with tumoral diseases. Anabolic agents acting on the bone include parathyroid hormone peptides, simvastatin, prostaglandin EP4 receptor antagonist, vitamin D, and strontium ranelate; anti-catabolic agents with bone action include compounds such as calcitonin, bisphosphonates, members of the tumor necrosis factor superfamily, and selective estrogen receptor modulators [7,8,9]. To prevent peri-implant bone loss and some adverse reactions reported in long-term oral treatment (hypocalcemia, mineralization disorders, gastrointestinal side effects, and osteomyelitis/osteonecrosis of the jaw, the most unpleasant side effect caused by systemic administration of bisphosphonates), a solution frequently studied in recent years is local treatment through drug delivery systems. Such a system must include a sufficient amount of active substance and have sustained release, allowing the desired therapeutic effect [10,11]. Orally administered CaFb is effective in alleviating symptoms of the physiological stress response, including mucosal inflammation, discomfort associated with osteoarthritis disorders and bone loss, and also in supporting cardiovascular health. Clinical studies have highlighted CaFb’s capacity to significantly modulate molecular markers associated with inflammatory mechanisms, primarily the increased serum levels of C-reactive protein [12,13,14].

Beyond these systemic anti-inflammatory and antioxidant effects, calcium fructoborate has also been investigated directly in the context of bioceramics and implant surface functionalization. In the present work, these biological properties are considered only as a rationale for selecting CaFb as an osteoactive candidate; the adsorption behavior on HAp/HApTi is instead governed by its physicochemical features (borate–di-fructose scaffold, multiple hydroxyl and oxygen donor groups) and is evaluated independently by docking, MD and adsorption–release experiments. Sol–gel and precipitation routes have been used to obtain hydroxyapatite nanopowders doped with CaFb, yielding bionanocomposites with appropriate phase composition, microstructure and in vitro bioactivity for potential use as osteosynthesis materials [15]. Moreover, CaFb has been applied as an adsorbed surface coating on titanium–hydroxyapatite (HApTi) implants, where in vivo studies in a rabbit femur model demonstrated improved osseointegration, reduced inflammatory response, and a favorable pattern of osteocalcin and osteopontin expression at the bone–implant interface compared with unmodified HApTi [16]. CaFb-containing HAp/Ti composites have also been proposed as platforms for adsorption-based loading and controlled release of antibiotics, supporting the concept that CaFb can be integrated into multifunctional, drug-eluting bioceramic systems [17]. These data indicate that CaFb should not be regarded solely as a dietary supplement, but also as a bioactive moiety capable of modulating the local tissue response when incorporated into calcium phosphate–based biomaterials. However, the mechanistic basis of its interaction with HAp/HApTi surfaces and its performance relative to clinically used osteotropic agents such as alendronate salts remain insufficiently characterized at the molecular level.

Although there is already an extensive experimental literature on bisphosphonate-coated implants, including alendronate- and zoledronate-modified titanium and calcium phosphate surfaces, these data do not fully clarify how such agents compare mechanistically with newer candidates such as CaFb or how different ligand chemistries translate into binding strength and release profiles on HAp versus HApTi substrates. Systematically reproducing all possible combinations of ligands, salt forms and substrate compositions in vitro and in vivo would require substantial time, cost and animal use. In this context, molecular docking and molecular dynamics simulations provide a rational in silico pre-screening tool that allows us to quantify and compare the stability of ligand–substrate complexes, identify key interaction patterns (hydrogen bonding, electrostatic and coordination interactions) at the interface, and generate testable hypotheses regarding adsorption capacity and release kinetics before undertaking extensive experimental work. In the present study, such in silico analyses are used not as a replacement for established experimental evidence, but as a complementary mechanistic approach to interpret and prioritize osteoactive functionalization strategies for HAp and HApTi implants.

From a surface chemistry perspective, stoichiometric hydroxyapatite (HAp) exposes Ca^2+^, PO_4_^3−^ and OH^−^ groups at the solid–liquid interface, leading to a highly polar, strongly hydrated surface dominated by Ca–O coordination sites and hydrogen-bond donors and acceptors. At physiological pH, the outermost HAp layer typically carries a net negative charge arising from phosphate groups, while surface calcium ions act as Lewis-acidic centers that can bind carboxylate, phosphate and hydroxyl functionalities of adsorbed molecules. In contrast, HAp–titanium (HApTi) composites combine this calcium phosphate phase with a metallic titanium component that is rapidly covered by a native TiO_2_/TiO_x_ layer. The resulting heterogeneous surface presents both Ca/PO_4_/OH sites and Ti–O/Ti–OH groups, with a different distribution of charge, coordination environments and Lewis acidity compared with pure HAp. These compositional and chemical differences are expected to influence the strength and geometry of ligand binding, particularly for polyfunctional molecules such as CaFb and bisphosphonate salts, and they provide the rationale for separately modeling ligand interactions with HAp and HApTi in our docking and molecular dynamics simulations [18]. At the nanoscale, adsorption on hydroxyapatite is strongly influenced by surface charge, Ca/P ratio and the specific crystallographic planes exposed to the solution. Stoichiometric HAp (Ca_10_(PO_4_)_6_(OH)_2_; Ca/P = 1.67) typically develops a slightly negative surface charge at physiological pH due to partial deprotonation of surface phosphate groups, while surface Ca^2+^ ions act as Lewis-acidic sites for coordination with carboxylate, phosphate, hydroxyl and amine groups of adsorbed molecules. Deviations from the ideal Ca/P ratio modify the density of Ca^2+^ and PO_4_^3−^ sites, thus altering the number and strength of potential binding sites for multidentate ligands such as bisphosphonates and CaFb. In addition, different crystallographic facets of HAp present distinct arrangements and densities of Ca^2+^ and PO_4_^3−^ at the interface, leading to facet-dependent adsorption energies and binding geometries that have been demonstrated in atomistic simulations and adsorption experiments. Consequently, both the global Ca/P ratio and the local distribution of charged groups on specific planes must be considered when interpreting ligand–HAp interactions, and they provide a physicochemical basis for the differences in docking scores, MD-derived stability and adsorption behavior observed in this study [19].

Periprosthetic osteolysis is fundamentally a local process that develops at the bone–implant interface, driven by wear particles, inflammatory mediators and dysregulated bone remodeling in the peri-implant region. While systemic antiresorptive or anti-inflammatory drugs can slow generalized bone loss, they do not selectively target the microenvironment adjacent to the implant and may be associated with systemic adverse effects when used long term. In contrast, bioactive surface coatings on metallic or calcium phosphate implants provide a means to modulate this local microenvironment directly, by promoting osteoblast activity, limiting inflammatory cell recruitment and delivering osteoactive agents at therapeutic concentrations precisely where bone–implant integration is required. Therefore, in the context of periprosthetic osteolysis, CaFb and alendronate salts are considered in this work not as systemic medicines, but as locally acting components of HAp/HApTi surface functionalization strategies designed to influence the balance between bone resorption and formation at the implant interface [20].

Previous computational work has already addressed the interaction of various ligands with hydroxyapatite surfaces. Density functional theory (DFT) and molecular dynamics (MD) simulations have been used to model the adsorption of several bisphosphonates on specific HAp facets, showing that binding affinity depends strongly on protonation state, side-chain structure and surface orientation [21]. Other MD studies have examined the adsorption of proteins and peptides, such as basic fibroblast growth factor or statherin-derived sequences, onto HAp, revealing a key role of electrostatic interactions and charged residues in anchoring biomolecules to Ca^2+^/phosphate-rich surfaces and stabilizing their conformation at the interface [22]. Collectively, these investigations demonstrate that atomistic simulations can rationalize and predict HAp–ligand binding behavior and are consistent with experimental evidence on bisphosphonate–bone mineral affinity [23,24]. However, to our knowledge, no computational study has yet explored the interaction of calcium fructoborate with HAp or HApTi, nor directly compared CaFb with different alendronate salt forms on both pure HAp and HAp–titanium composite surfaces while linking the predicted binding strengths to adsorption–release profiles. The present work is therefore intended to extend existing HAp–ligand modeling to a CaFb/HApTi system that has already shown promising in vivo performance, and to place bisphosphonate-functionalized implants and CaFb-based coatings within a unified mechanistic framework.

To provide a clearer framework for comparing these ligands, the present work was designed to integrate in silico and experimental analyses in order to mechanistically evaluate how calcium fructoborate, sodium alendronate and calcium alendronate interact with HAp and HApTi surfaces. The aim is to determine which compound offers the most stable binding configuration and the most sustained release behavior, thereby informing future strategies for osteoactive implant surface engineering.

Therefore, the present study aimed to comparatively evaluate calcium fructoborate, sodium alendronate and calcium alendronate as potential osteoactive agents for the functionalization of HAp and HApTi implants. We combined molecular docking and molecular dynamics simulations with adsorption–release experiments to identify the ligand that exhibits the most stable interaction with HAp/HApTi substrates, the highest loading capacity and the most favorable release profile for future in vivo applications. 

## 2. Materials and Methods

### 2.1. Molecular Docking and Dynamic Simulations

To guide our selection of the active principle to be adsorbed on the surface of HAp and HApTi samples, we conducted a comparative theoretical study between CaFb, sodium alendronate, and calcium alendronate. The ligand structures, HAp, and HApTi were geometrically optimized using the Gaussian 09 software (Gauss View 16 interface) through the DFT/B3LYP/6-31G method. HAp and HApTi were represented by charge-neutral cluster models intended to approximate the local coordination environment of hydroxyapatite-based surfaces rather than fully periodic slabs. The HAp cluster was constructed from stoichiometric hydroxyapatite (Ca_10_(PO_4_)_6_(OH)_2_; Ca/P = 1.67), preserving the characteristic arrangement of Ca^2+^, PO_4_^3−^ and OH^−^ groups at the outer shell. Dangling bonds at the cluster boundary were capped with hydroxyl or hydrogen atoms to remove unrealistic undercoordinated sites and to maintain overall electroneutrality. Surface phosphate groups were assigned protonation states consistent with a near-physiological pH environment, yielding a slightly negative net surface character with exposed Ca^2+^ acting as Lewis-acidic sites. For the HApTi model, titanium atoms and their coordinating oxygen atoms were introduced into one region of the HAp cluster to mimic the experimentally used HApTi composite, in which a titanium phase covered by oxo/hydroxo species coexists with the calcium phosphate matrix. As a result, the outermost shell of the HApTi cluster contains both Ca/PO_4_/OH moieties and Ti–O/Ti–OH groups, reflecting the heterogeneous surface chemistry of the composite. Because these models are finite clusters, they do not correspond to a single well-defined crystallographic plane; instead, they provide a generic representation of low-index Ca/PO_4_/OH- and Ti–O/Ti–OH-terminated surface environments suitable for comparative docking and MD simulations of ligand binding. For all ligands, protonation state and total formal charge were assigned to approximate physiological pH (7.4), based on reported pK_a values and PubChem annotations, and the dominant species at this pH was used for docking and MD. Alendronate was modeled with deprotonated phosphonate groups and a protonated terminal amine, yielding an overall negatively charged bisphosphonate; sodium and calcium were treated as counterions and were not explicitly included in the docking step. Calcium fructoborate was considered in its neutral complex form, preserving the borate–di-fructose coordination, and partial atomic charges for all ligands in these protonation states were generated with AutoDockTools (Gasteiger–Marsili scheme). Molecular docking analysis was carried out in triplicate using the Autodock 4.2.6 software along with the AutoDockTools molecular viewer. In the mapping stage, we set the mapping box to 10X10X10 at a distance of 0.5 angstroms from the target center. In the docking stage, we selected the Lamarckian Genetic Algorithm with a number of 30 runs. The images of the target-ligand complexes were visualized using Discovery Studio Visualization 2020 (Biovia, San Diego, CA, USA) [25]. The binding free energy (ΔG_binding) in AutoDock 4.2 was calculated as the sum of van der Waals (ΔG_vdW_), hydrogen bonding (ΔG_hbond_), electrostatic (ΔG_electrostatic_), desolvation (ΔG_desolvation_), and torsional (ΔGt_orsional_) contributions. Negative ΔG values indicate spontaneous and stable ligand–target interactions:

ΔG_binding_ = ΔG_vdW_ + ΔG_hbond_ + ΔG_electrostatic_ + ΔG_desolvation_ + ΔG_torsional_

After molecular docking calculations, top-scoring ligand-receptor complexes were subjected to 100 ns all-atom MD simulations to investigate the ligand–substrate interactions in more detail and to determine the binding free energies accurately. In MD simulations Sibiolead software (2025 release)was used. For the organic ligands, bonded and non-bonded parameters were taken from the AMBER14 force field, while ligand topologies were generated with SWISSPARAM. For the inorganic HAp and HApTi clusters, AMBER-compatible non-bonded parameters (Lennard–Jones terms and partial charges) were assigned by mapping Ca, P, O and H atoms to existing AMBER ion and phosphate oxygen types and adjusting charges to reproduce the stoichiometric Ca_10_(PO_4_)_6_(OH)_2_ composition and overall electroneutrality of the clusters. Titanium atoms and their coordinating oxygen atoms in HApTi were described using oxide-like Ti–O atom types with locally charge-balanced Ti–O/Ti–OH environments. During MD simulations, the inorganic cores were kept position-restrained, so that they acted as rigid adsorption templates, and the MD results are therefore interpreted in a semi-quantitative manner, focusing on relative trends between ligands on the same surface model rather than on absolute adsorption energies. After neutralization of the system, energy minimization was performed for each complex by employing the steepest descent minimization algorithm with 5000 steps. After performing 200 ps NVT and NPT ensemble equilibrations, MD simulations were performed in a dodecahedron simulation box for 100 ns at 1 bar and 300 K reference pressure and temperature. Simulations were performed with the AMBER14 force field and SPC water model. A real-space cutoff of 1.0 nm was applied for short-range van der Waals and electrostatic interactions, and long-range electrostatics were treated with the particle-mesh Ewald (PME) method. All covalent bonds involving hydrogen atoms were constrained using the LINCS algorithm, allowing the use of a 2 fs integration time step. Isothermal–isobaric (NPT) production simulations were carried out using a velocity-rescale thermostat (τ_T = 0.1 ps) to maintain the temperature at 300 K and a Parrinello–Rahman barostat (τ_P = 2.0 ps, compressibility 4.5 × 10^−5^ bar^−1^) to control pressure at 1 bar. Periodic boundary conditions were applied in all three dimensions in a dodecahedral simulation box with at least 1.0 nm of SPC water padding between the target–ligand complex and the box boundaries.

Docking calculations for each ligand–substrate pair were performed in triplicate as three independent runs with identical settings, and the corresponding binding energies were summarized as mean ± standard deviation. For MD simulations, structural descriptors such as ligand RMSD (relative to the initial docked pose) and radius of gyration were recorded every 10 ps over the 100 ns trajectories. After discarding the initial 50 ns as equilibration, time-averaged values, SD and the observed ranges were computed from the remaining production frames for each complex. In addition, root mean square fluctuation (RMSF) profiles were computed to characterize local flexibility. RMSF values were calculated for ligand heavy atoms and for the heavy atoms of the outermost HAp/HApTi surface layer over the production phase of the trajectories, after least-squares fitting to the inorganic cluster. These analyses allowed us to compare ligand flexibility relative to the essentially rigid, position-restrained substrates.

HAp and HApTi substrates were modeled as finite, charge-neutral clusters representing the local coordination environment of hydroxyapatite-based surfaces. Atom types and partial charges for Ca, P, O and H were assigned using AMBER-compatible parameters and adjusted to preserve the stoichiometric Ca_10_(PO_4_)_6_(OH)_2_ composition and overall electroneutrality of the HAp cluster. For the HApTi model, titanium atoms and their coordinating oxygen species were introduced into the outer region of the HAp cluster and described using oxide-like Ti–O/Ti–OH atom types to reflect the heterogeneous surface chemistry of the composite. During molecular dynamics simulations, positional restraints were applied to the inorganic substrate atoms so that the substrates acted as rigid adsorption templates, allowing the analysis to focus on ligand–surface interactions rather than bulk lattice relaxation.

Because the HAp and HApTi models used in this study are finite, charge-neutral clusters rather than periodic slabs, the computed binding energies and dynamic stability descriptors should be interpreted qualitatively. These models capture local coordination environments but do not account for long-range periodicity, facet-specific reconstruction or microstructural heterogeneity. Therefore, the docking and MD results are intended to identify relative trends between ligands rather than to represent absolute adsorption energetics.

### 2.2. Preparation of HAp and HApTi Implants by Powder Metallurgy

HAp and HApTi implants were obtained by powder metallurgy. Briefly, hydroxyapatite (HAp, Ca_5_(PO_4_)_3_(OH), Merck, Darmstadt, Germany, ~200 nm) powder was first calcined in air at 900 °C for 1 h, then mixed with or without titanium hydride (TiH_2_, Merck, ~100 µm) in a planetary ball mill (for HApTi, 75 wt% HAp and 25 wt% TiH_2_; ball-to-powder ratio 2:1 in ethanol, 30 min). The dried powder mixtures were uniaxially cold-compacted at 120 MPa in a 10 mm die and sintered in a two-step schedule under flowing argon, with a short hold at 900 °C followed by a 10 h dwell at 800 °C, to obtain dense HAp and HApTi disc-shaped implants.

### 2.3. Surface Adsorption and Release of Salts

To investigate the potential of surface functionalization with osteoactive agents, selected sintered HAp/HApTi implants were subjected to adsorption–release experiments using calcium fructoborate, sodium alendronate, and calcium alendronate. The HApTi biocomposite discs were prepared as described above and were thoroughly rinsed and dried at room temperature prior to functionalization. All samples were weighed before and after the adsorption step in order to determine the mass of adsorbed compound by gravimetry and to express the loading as mass per unit surface area.

#### Adsorption and Release of Calcium Fructoborate

For the CaFb experiments, a standard CaFb material (VDF FutureCeuticals, Momence, IL, USA; purity 2.7% expressed as boron, verified by nuclear magnetic resonance) was used. Chromatographic-grade solvents and reagents, including 2-propanol, ethanol (99%), and ultrapure water (LiChrosolv^®^, Merck, Darmstadt, Germany), were employed for sample preparation and analysis. A CaFb stock solution was prepared by dissolving 0.30 g CaFb in 10 mL distilled water under gentle stirring until complete dissolution. HApTi discs were fully immersed in this solution in sealed glass vials and maintained at room temperature for 24 h to allow adsorption onto the implant surface. The specimens were then removed, gently blotted to remove excess solution, dried at room temperature for 24 h, and re-weighed. The difference between the final and initial masses was taken as the amount of CaFb adsorbed.

The release of CaFb from the HApTi surfaces was assessed by immersing the adsorbate-loaded specimens in 10 mL simulated synovial test fluid (SSTF) contained in tightly sealed vials. The vials were kept at 37 ± 0.5 °C for 15 days. At regular time intervals, 0.5 mL aliquots of the release medium were withdrawn and immediately replaced with equal volumes of fresh SSTF to maintain sink conditions and constant volume. The concentration of CaFb in the collected samples was quantified by high-performance thin-layer chromatography (HPTLC) using calibration curves constructed from CaFb standards prepared in the same medium. To confirm the chemical identity of the released compound, one representative HPTLC band corresponding to CaFb was eluted directly into a mass spectrometer and analyzed by electrospray ionization mass spectrometry in negative mode. The mobile phase consisted of methanol and 10 mM ammonium acetate (9:1, *v*/*v*); the probe temperature was set to 450 °C, the capillary voltage to 0.8 kV, and the cone voltage to 25 V, allowing the acquisition of a characteristic mass spectrum of CaFb and its fragments.

For the alendronate studies, pharmaceutical-grade sodium alendronate and calcium alendronate were used. Dried HApTi discs were weighed and then immersed in freshly prepared aqueous alendronate solutions. Each salt was dissolved at a concentration of 0.30 mg/10 mL in 0.05–0.10 M borate buffer, adjusted to pH 9.0–9.5 to ensure complete dissolution of the bisphosphonate. Individual discs were placed in 10 mL of sodium alendronate or calcium alendronate solution in sealed glass vials and incubated at room temperature for 48 h without agitation. After this step, the samples were removed, briefly rinsed with a small volume of buffer to eliminate loosely bound material, dried on filter paper, and left at room temperature for an additional 48 h. The discs were then re-weighed, and the difference between the final and initial masses was attributed to surface-adsorbed alendronate.

Release profiles for sodium alendronate and calcium alendronate were obtained by immersing the functionalized discs in 5–10 mL of ultrapure water or SSTF at 37 ± 0.5 °C. At predefined time points (typically 1, 6, 12, 24, 48, and 72 h, followed by days 6, 9, 12, and 14), 0.5 mL aliquots of the supernatant were collected and immediately replaced with equal volumes of fresh medium. Quantification of the released alendronate was performed by reversed-phase high-performance liquid chromatography (HPLC) after pre-column derivatization with 9-fluorenylmethyl chloroformate (FMOC-Cl). Briefly, each sample aliquot was mixed with borate buffer and 30 mM FMOC-Cl in methanol, allowed to react for 5 min at room temperature, and then injected into a C18 column (250 × 4.6 mm, 5 µm). The FMOC-alendronate derivatives were eluted isocratically using an acetonitrile–aqueous buffer mobile phase at a flow rate of 1.0 mL/min and detected by UV absorbance at approximately 260–270 nm. Calibration curves constructed from derivatized sodium and calcium alendronate standards were used to determine the concentrations in the release samples and to calculate the cumulative amount released over time. All surface adsorption and release experiments were performed in triplicate for each condition; the values reported in the tables correspond to the mean ± standard deviation (SD) of the three independent measurements.

## 3. Results

The primary purpose of the results presented in this section is to comparatively screen and rank the investigated ligands under consistent computational and experimental conditions. Accordingly, the reported metrics are used to establish relative differences in adsorption, interfacial stability, and retention behavior rather than absolute performance values.

## 4. Discussion

The in silico findings complement previous experimental research on surface adsorption-based functionalization of HApTi composites, where enhanced drug adhesion and controlled release were demonstrated [26,27]. The theoretical results indicate that calcium fructoborate (CaFb) forms multiple hydrogen bonds and metal–acceptor interactions with HAp and HApTi surfaces, suggesting a strong and stable ligand–substrate affinity. Such a binding pattern supports the experimentally observed potential of CaFb-functionalized bioceramics to ensure sustained drug delivery and improved cellular compatibility. These computational insights therefore provide a molecular-level explanation for the favorable physicochemical and biological behavior previously reported for adsorbed coatings.

Recent literature also supports that implant surface functionalization with osteoactive molecules is a promising approach for improving osseointegration and preventing periprosthetic osteolysis [28,29]. Studies performed on animal models have shown that surface deposition of bioactive agents on metallic or ceramic substrates allows a controlled and sustained drug release, enhancing cellular adhesion and osteoblastic differentiation [30]. CaFb has been reported in the literature to exhibit anti-inflammatory and antioxidant effects in systemic and local contexts [31]; however, these effects were not evaluated in the present study and are not inferred from the in silico or adsorption–release results. Histological and immunohistochemical findings from previous in vivo studies have reported enhanced expression of osteogenic markers, including osteocalcin and osteopontin, together with improved peri-implant bone parameters for CaFb-containing coatings [32,33]. Such biological outcomes were not evaluated in the present in silico or adsorption–release study.

Integrating the theoretical and experimental findings demonstrates that molecular modeling can serve as an efficient preliminary stage for screening active principles with high affinity toward bioceramic materials. Such computational approaches reduce the need for extensive early-phase testing, allowing for optimization of both composition and deposition method prior to in vitro and in vivo validation [7,34,35,36].

Analyzing the data provided by PubChem, we observed that CaFb has 12 hydrogen bond donors and 26 hydrogen bond acceptors, while sodium alendronate has 5 hydrogen bond donors and 8 hydrogen bond acceptors, and calcium alendronate has 10 hydrogen bond donors and 16 hydrogen bond acceptors. Considering the polar nature of HAp and HApTi substrates, we assumed that the adsorption of CaFb would be favored by the possibility of forming a greater number of hydrogen bonds, compared to the salts of alendronic acid. In the literature, we found numerous studies involving the functionalization of implants with bisphosphonates, so we decided to choose CaFb as the active principle, since this research direction is much less explored.

From the binding energy values, we can observe that the formation of substrate-sodium alendronate complexes is energetically favored, being a spontaneous process (Table 1, Table 2, Table 3 and Table 4). The two types of substrates do not form spontaneous complexes with calcium alendronate (having positive values of binding energy), with hydrogen bonds and metal–acceptor type interactions occurring between the substrate and ligand. From the results obtained, we can see that between both substrates (HAp, HApTi) and sodium alendronate, hydrogen bonds and metal–acceptor type interactions are formed (Table 2 and Table 4). The HApCaFb and HApTiCaFb complexes form spontaneously (Table 1 and Table 3), being stabilized through hydrogen bonds, metal–acceptor interactions, and acceptor–donor interactions (Table 2 and Table 4). From the in silico results obtained, we observe that the formation of complexes between substrates-sodium alendronate and substrates-CaFb occurs spontaneously, and the complexes are stabilized through classical hydrogen bonds, non-classical hydrogen bonds, acceptor–donor interactions, and metal–acceptor interactions. A greater stability of substrates with CaFb is highlighted, compared to sodium alendronate, supported by a greater number of non-covalent interactions.

An apparently paradoxical result of the in silico analysis is that calcium alendronate exhibits positive binding free energies on both HAp and HApTi, despite forming a relatively large number of hydrogen bonds at the interface. This behavior can be rationalized by considering the full electrostatic context rather than the hydrogen-bond count alone. In our models, the HAp/HApTi surfaces carry a net negative character due to exposed phosphate groups, while calcium alendronate presents a highly localized distribution of negative charge around the phosphonate moieties, only partially compensated by the coordinated Ca^2+^ counterion. When this internally neutralized complex approaches a likewise Ca^2+^/PO_4_^3−^-rich surface, the geometry does not allow an optimal alignment between the ligand’s phosphonate oxygens and surface Ca^2+^ sites; instead, significant regions of like-charged groups remain in close proximity. The resulting electrostatic repulsion, combined with the desolvation penalty associated with stripping water molecules from both the ligand and the negatively charged surface, can outweigh the favorable enthalpic contribution of hydrogen bonding, leading to a net positive ΔG. By contrast, in the sodium alendronate systems, the more weakly bound Na^+^ counterions are readily solvated, allowing the deprotonated bisphosphonate moiety to engage more directly with surface Ca^2+^ centers and to adopt binding orientations in which electrostatic attraction dominates over repulsion. These considerations highlight that hydrogen-bond counts alone are insufficient to predict binding affinity and that the detailed balance between electrostatic attraction, repulsion and desolvation is critical for understanding the less favorable binding of calcium alendronate compared with CaFb and sodium alendronate. High RMSD values in subsequent molecular dynamics simulations suggest that the ligand-target complex is unstable, with the ligand showing significant conformational mobility. This implies that the initial binding pose from docking may not form a stable complex in a dynamic environment like physiological conditions. The high RMSD values indicate that the binding interaction captured by docking is not energetically optimal, leading to larger deviations in the ligand’s position relative to the target during the simulation. Hence, the strong binding energy in molecular docking and the high RMSD values in molecular dynamics simulations suggest a less stable interaction between the ligand and receptor, possibly due to weak binding forces, significant conformational changes, or both [21,22,23].

RMSD of ligand compared to the position of the target was monitored for each complex to investigate how well the binding pose was preserved during the MD simulation. High RMSD values would be correlated to significant instability, being related to changes within the conformation of the complexes investigated.

For HAp–sodium alendronate complex the simulation data indicates that the RMSD values stabilize quickly within the initial phase of the simulation, maintaining a narrow band predominantly below 0.5 nm, mean RMSD value is 0.27 ± 0.17 nm. This trend demonstrates the conformational stability of the HAp–sodium alendronate complex, with no significant structural deviations observed that would suggest instability. The mean RMSD value observed throughout the simulation period suggests a tightly regulated conformational integrity of the complex. The lack of high RMSD peaks further implies that the complex does not undergo large-scale structural rearrangements but remains consistently close to its initial structure. Notably, after the equilibration period, the RMSD settles into a consistent pattern with only minor fluctuations. These are indicative of the dynamic nature of molecular interactions but do not detract from the overall stability of the complex. The low RMSD values are suggestive of the complex’s potential.

The RMSD for the HAp–calcium alendronate complex shows variations within a wide spectrum, ranging from 0.35 to 1.1 nm, RMSD mean value being 0.72 ± 0.28 nm. This indicates that the structure of the complex fluctuates within this range, suggesting some degree of flexibility or conformational variability. RMSD is a measure of the average distance between atoms of superimposed molecules, and in this case, it reflects the degree of deviation or movement within the HAp–calcium alendronate complex over time or in different conditions.

**Figure 1:**
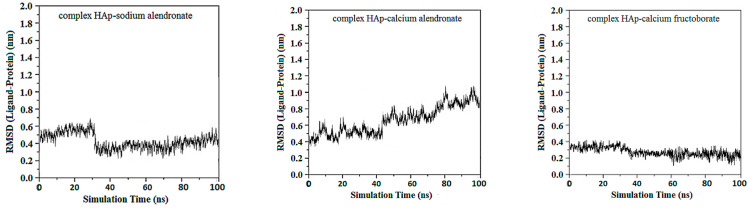
RMSD ligand–HAp substrate.

Over the simulation period for the HAp-CaFb complex, the RMSD fluctuates primarily between approximately 0.15 nm and 0.4 nm, suggesting that after an initial equilibration phase, the complex achieves a relatively stable conformation. The graph does not indicate significant conformational changes that would suggest instability of the ligand-target complex. This stability is particularly evident after the 20 ns mark, where the RMSD values appear to level off, indicating that the complex reaches a dynamic equilibrium. The mean RMSD value calculated from the data points is 0.207 nm, with a standard deviation of 0.190 nm. These results imply that the HAp-CaFb complex maintains a moderately consistent conformation with some flexibility over the course of the simulation. The modest standard deviation supports the premise of conformational consistency, although the presence of any minor deviations from the mean RMSD could be attributed to normal thermal fluctuations within the simulated system for high-affinity binding and structural fidelity in its intended biological context (Figure 1).

From the first graph of HApTi-sodium alendronate complex, the mean RMSD value is 0.3 ± 015 nm, start low but then display a more pronounced variability, although without a consistent upward or downward trend. This fluctuation might indicate that while the complex exhibits dynamic behavior, it alternates between several stable conformations or binding modes over the course of the simulation.

For HApTi-calcium alendronate complex the RMSD values of this complex exhibit a gradual increase over approximately 40 ns simulation period, suggesting that the complex may be experiencing conformational changes or has not fully stabilized. The upward trend implies that the ligand may be exploring different binding modes or conformations on the HApTi surface, RMSD value is 0.72 ± 0.38 nm.

**Figure 2:**
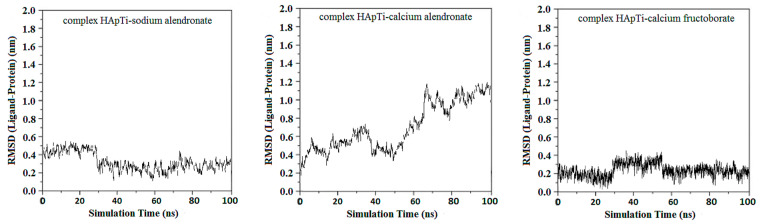
RMSD ligand–HApTi substrate.

The third graph of HApTi-CaFb complex shows relatively low RMSD fluctuations throughout the simulation 0.26 ± 0.14 nm, indicating that the ligand-target complex is relatively stable. The minor fluctuations around the baseline suggest a steady interaction without significant conformational changes, which could mean that the ligand maintains a stable binding pose (Figure 2). Results showed that, mainly hydrogen bonds (classical and non-classical) and some other types of interactions (metal–acceptor, acceptor–donor) take part in the stabilization of the complexes.

RMSF analysis further supported the RMSD-based assessment of conformational stability. For both HAp and HApTi, the restrained inorganic clusters showed only minimal atomic fluctuations (RMSF values generally < 0.05 nm), confirming that the substrates behaved as rigid adsorption templates throughout the simulations. In contrast, ligand RMSF profiles revealed clear differences between the three compounds. CaFb and sodium alendronate displayed uniformly low fluctuations of the heavy atoms directly involved in binding, with most RMSF values in the interfacial region typically below 0.15 nm and only modest increases up to ~0.18–0.20 nm at terminal, solvent-exposed groups, consistent with the preservation of a well-defined binding pose over time. By comparison, calcium alendronate exhibited higher RMSF values across larger portions of the molecule, with many interfacial atoms frequently approaching or exceeding 0.20–0.25 nm, indicating greater conformational mobility at the interface and a tendency to explore multiple binding orientations. These RMSF patterns agree with the RMSD trends and docking energies, reinforcing the picture of more stable complexes for CaFb (and, to a lesser extent, sodium alendronate) and a less stable, more dynamic interaction for calcium alendronate.

The adsorption data in Table 5 show a clear dependence of loading capacity both on the type of adsorbed salt and on the implant composition (HAp vs. HApTi). For both substrates, calcium fructoborate exhibits the highest adsorbed amount and percentage, followed by sodium alendronate and, finally, calcium alendronate. On HAp, the percentage of salt adsorbed decreases from 6.04% for CaFb to 5.29% for sodium alendronate and 4.21% for calcium alendronate. A similar hierarchy is observed on HApTi, where the adsorbed fraction reaches 9.15% for CaFb, 7.38% for sodium alendronate and 6.03% for calcium alendronate. This trend suggests that CaFb has a higher affinity for both ceramic and biocomposite surfaces, most likely due to its larger number of functional groups capable of hydrogen bonding and electrostatic interactions with surface hydroxyl and phosphate groups, as also indicated by the in silico analysis.

Comparison between the two implant types further indicates that the HApTi biocomposite provides a more favorable substrate for salt adsorption than pure HAp. For all three active principles, both the absolute amount adsorbed and the corresponding percentage are higher on HApTi than on HAp. This enhancement may be attributed to differences in microstructure and surface chemistry introduced by the titanium phase, which can increase surface roughness, create additional adsorption sites and modify local charge distribution at the interface.

**Figure 3:**
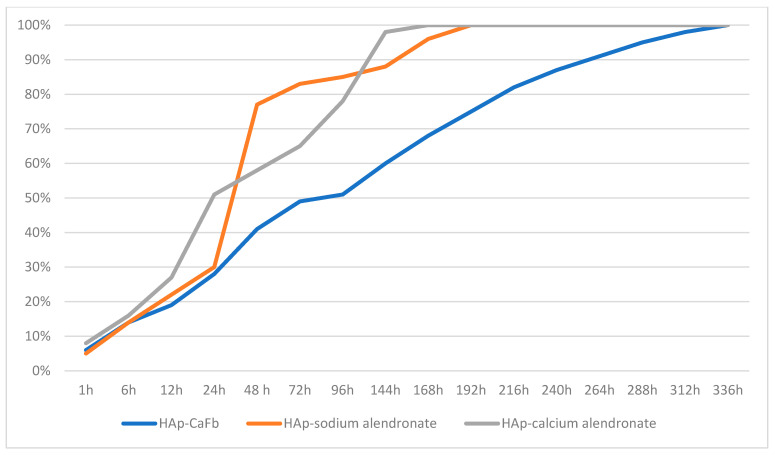
Time-dependent cumulative release of CaFb, sodium alendronate, and calcium alendronate from HAp samples.

**Figure 4:**
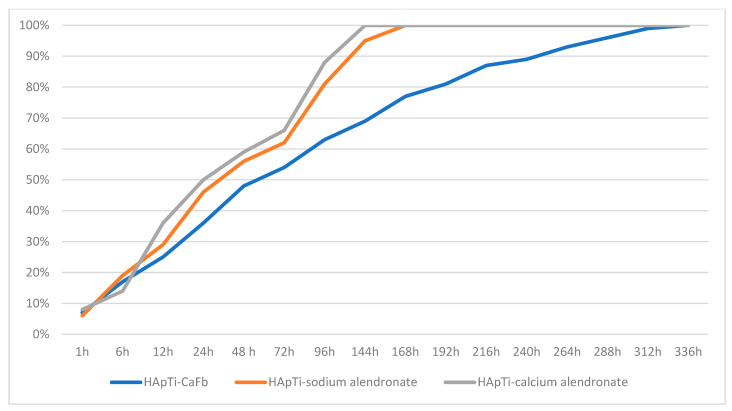
Time-dependent cumulative release of CaFb, sodium alendronate, and calcium alendronate from HApTi samples.

Balosache et al. previously observed that surface loading of alendronate onto HAp specimens by adsorption is not an optimal functionalization strategy, since the drug was almost completely released into the medium within the first two days, whereas a slow, prolonged release is desirable for sustained therapeutic action. To quantify the amount of CaFb released over two weeks, we constructed a CaFb calibration curve (R = 0.996152). The CaFb release profile showed a rapid initial release during the first 75 h, followed by an approximately linear release phase up to 14 days (Figure 3 and Figure 4). In addition to CaFb, the cumulative release profiles of the two alendronate salts from HAp and HApTi substrates (Figure 3 and Figure 4) reveal a markedly faster and more pronounced release. For both types of implants, calcium alendronate exhibits the steepest initial slope, followed closely by sodium alendronate, indicating a pronounced burst release during the first days of immersion. A major fraction of the adsorbed alendronate is liberated within the first week, after which the curves rapidly approach a plateau, consistent with the weaker and less stable interaction between the bisphosphonate moieties and the ceramic surface compared with CaFb. These observations are in line with previous reports showing that alendronate adsorbed onto HAp is almost completely eluted from the substrate within the first 48 h, making this functionalization strategy suboptimal when a long-term, sustained release is required.

Extended surface characterization techniques such as FTIR or SEM were not included in the present study, as the primary objective was to quantify adsorption capacity and release kinetics. Nonetheless, we acknowledge that such analyses could provide additional insight into surface chemistry and morphology, and they will be considered in future work.

When comparing HAp with HApTi, the overall shape of the release curves for both alendronate salts is similar, but the HApTi composite tends to slightly moderate the burst effect and extend the release phase over a longer period. This behavior may be attributed to the different microstructure and surface chemistry of the HApTi biocomposite, which can provide additional adsorption sites and diffusion pathways. Nevertheless, in all cases the alendronate salts display a substantially shorter release time than CaFb, which shows a slower initial release and a more linear, prolonged liberation over the entire 14-day observation period. Taken together, these results support the notion that CaFb establishes a more stable interaction network with the HAp/HApTi matrices than alendronate, in agreement with the in silico prediction of stronger substrate–ligand complexes for CaFb, and they highlight CaFb as a more suitable candidate for long-term osteoactive functionalization of HAp- and HApTi-based implants.

These findings are in very good agreement with the substrate–ligand stability predicted in our in silico analysis, where a higher number of HAp/HApTi–CaFb interactions, compared with HAp/HApTi–alendronate interactions, can account for the more sustained release of CaFb into the medium.

It is important to emphasize that the present work addresses periprosthetic osteolysis from the perspective of surface functionalization, not systemic pharmacotherapy. The role of CaFb and alendronate salts in our design is to act as locally active components of HAp/HApTi coatings, where their binding strength, loading capacity and release profile may modulate the immediate peri-implant microenvironment. Any potential benefit on periprosthetic bone preservation would thus arise from these localized effects at the coating–bone interface and will need to be confirmed in dedicated in vivo models of osteolysis.

The interaction profile of CaFb suggests that its adsorption on HAp/HApTi could modulate the local surface charge distribution via Ca^2+^ coordination and multiple hydroxyl/oxygen donor groups, which may in turn favor the adsorption of serum proteins and the adhesion of osteoblasts at the interface. Previous experimental studies on boron-containing biomaterials have reported osteogenic responses in association with BMP- and Runx2-related signaling pathways; these observations provide a possible biological context for CaFb-based coatings but are not directly assessed in the present work. Our docking, MD, and adsorption–release results do not constitute direct evidence for BMP- or Runx2-related activity. Although these biological pathways provide a plausible mechanistic background, they were not investigated experimentally in this work. Consequently, any inference regarding osteogenic or anti-inflammatory signaling should be regarded as speculative and will require confirmation through dedicated in vitro and in vivo studies. Its incorporation into titanium–hydroxyapatite composites aligns with the current push toward multifunctional implants capable of both structural and biological performance.

Several limitations of the present work should be acknowledged. First, the docking scores and MD-derived metrics (RMSD, RMSF, estimated ΔG) provide only a semi-quantitative description of interfacial stability under the specific model assumptions used here (finite HAp/HApTi clusters, SPC water, AMBER-compatible parameters for the inorganic cores) and are not designed to predict biological outcomes directly. Second, our simulations capture short- to medium-time scale adsorption events in an idealized aqueous environment, whereas in vivo osseointegration and osteolysis are governed by complex cell–matrix interactions, remodeling dynamics and systemic factors that are beyond the scope of this study. Thus, the computational results should be viewed as hypothesis-generating and as a tool to prioritize ligands for experimental testing, rather than as standalone evidence of biological osteogenic or anti-osteolytic activity. Accordingly, the reported docking, MD, and adsorption–release results are interpreted in a comparative manner to identify relative trends between ligands under identical conditions, rather than as absolute thermodynamic quantities. All molecular dynamics analyses were performed using a single production trajectory per ligand–substrate system; accordingly, the reported standard deviations for RMSD, RMSF and related descriptors reflect time-series variability within each trajectory rather than statistical uncertainty derived from independent replicate simulations.

Building on the present in silico and adsorption–release data, future work will focus on refining CaFb-based surface modification strategies for HAp and HApTi substrates. Beyond simple adsorption, we plan to compare alternative coating techniques such as sol–gel deposition, hydrothermal treatments and layer-by-layer or polyelectrolyte-assisted assembly, in order to control coating thickness, homogeneity and long-term stability under simulated physiological loading. In parallel, in vitro bioactivity studies will be undertaken using osteoblast-like cells and mesenchymal stem cells to assess cell adhesion, proliferation, alkaline phosphatase activity, osteogenic marker expression (osteocalcin, osteopontin, Runx2) and mineralized matrix formation on CaFb- and alendronate-functionalized HAp/HApTi surfaces. Additional experiments will evaluate the inflammatory profile and oxidative stress markers in co-culture models that better mimic the peri-implant microenvironment. These investigations are expected to clarify how specific coating protocols and release profiles translate into osteoconductive and potentially anti-osteolytic effects at the bone–implant interface.

## 5. Conclusions

In this work, a combined in silico and experimental approach was employed to compare the interfacial behavior of calcium fructoborate, sodium alendronate, and calcium alendronate on HAp and HApTi substrates. Molecular docking and molecular dynamics simulations were used to assess ligand–substrate interaction patterns and interfacial stability, while adsorption–release experiments provided quantitative data on surface loading and retention behavior.

Under the selected computational models and experimental conditions, calcium fructoborate exhibited the most favorable interfacial characteristics on both HAp and HApTi, including lower binding free energies, reduced conformational fluctuations during molecular dynamics simulations, and higher adsorption capacity compared with the alendronate salts. Sodium alendronate showed intermediate adsorption and retention behavior, whereas calcium alendronate displayed less favorable binding and greater interfacial instability.

Release studies further revealed distinct retention trends among the investigated ligands. Calcium fructoborate demonstrated a more sustained release profile over the investigated period, while both alendronate salts exhibited a pronounced initial burst followed by rapid plateauing, consistent with their weaker interfacial retention.

Overall, the present results define comparative trends in ligand adsorption, interfacial stability, and retention on HAp- and HApTi-based surfaces within the applied in silico models and adsorption–release framework. These findings are intended to support the rational selection of candidate surface functionalization molecules for further experimental validation, without implying biological efficacy or clinical performance.

## Figures and Tables

**Table 1:** Free binding energy (ligand-target) (kcal/mol) and 3D image of the ligand–substrate interactions. The target is represented by spheres of different colors, depending on the type of constituent atom, and the ligand by lines. Classical hydrogen bonds are represented by dotted green lines, non-classical hydrogen bonds by dotted gray lines, and metal–acceptor interactions by dotted white lines.

The Type of Complex	Free Binding Energy (kcal/mol) (Mean ± SD)	3D Image of the Ligand–Substrate Interaction
HAp–sodium alendronate	−1.22 ± 0.00	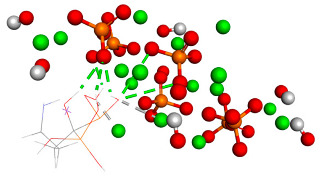
HAp–calcium alendronate	2.423 ± 0.006	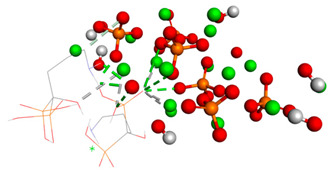
HAp–CaFb	−1.31 ± 0.00	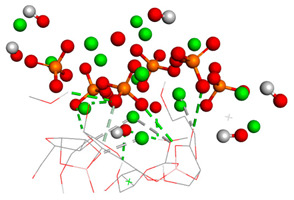

**Table 2:** The types of bonds established between ligand–substrate. The table expresses in angstroms the distance of classical hydrogen bonds, non-classical hydrogen bonds, and metal–acceptor interactions.

The Type of Complex	The Type of Ligand–Substrate Interactions
HAp–sodium alendronate	6 classical hydrogen bonds (2.04 Å; 2.06 Å; 2.18 Å; 2.37 Å; 3.33 Å; 3.39 Å) 2 metal–acceptor bonds (2.22 Å; 2.62 Å)
HAp–calcium alendronate	7 classical hydrogen bonds (2.0 Å; 2.14 Å; 2.15 Å; 2.49 Å; 2.78 Å; 3.08 Å; 3.23 Å) 6 non-classical hydrogen bonds (2.19 Å; 2.31 Å; 2.38 Å; 3.29 Å; 3.32 Å; 3.57 Å)
HAp–CaFb	8 classical hydrogen bonds (2.19 Å; 2.31 Å; 2.33 Å; 2.54 Å; 2.55 Å; 2.86 Å; 2.87 Å; 3.26 Å) 5 metal–acceptor bonds (2.45 Å; 2.83 Å; 2.88 Å; 3.38 Å; 3.43 Å)

**Table 3:** Free binding energy (ligand-target) (kcal/mol) and the 3D image of the ligand–substrate interactions. The target is represented by spheres colored differently according to the type of constituent atom, and the ligand by lines. Classical hydrogen bonds are represented by dotted green lines, non-classical hydrogen bonds by dotted gray lines, acceptor–donor interactions by dotted red lines, and metal–acceptor interactions by dotted white lines.

The Type of Complex	Free Binding Energy (kcal/mol) (Mean ± SD)	3D Image of the Ligand–Substrate Interaction
HApTi–sodium alendronate	1.537 ± 0.006	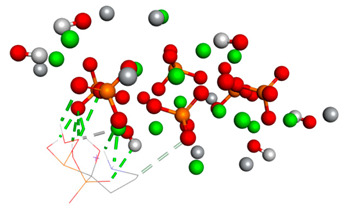
HApTi–calcium alendronate	+1.90 ± 0.00	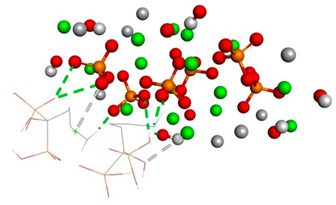
HApTi–CaFb	−1.63 ± 0.00	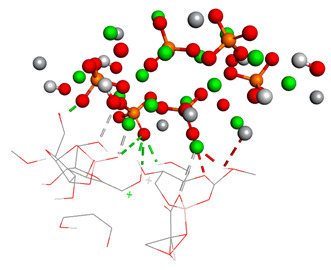

**Table 4:** The types of bonds established between ligand and substrate. The distances of classical hydrogen bonds, non-classical hydrogen bonds, and metal–acceptor interactions are expressed in angstroms in the table.

The Type of Complex	The Type of Ligand–Substrate Interactions
HApTi–sodium alendronate	7 classical hydrogen bonds (2.03 Å; 2.07 Å; 2.13 Å; 2.18 Å; 2.30 Å; 2.61 Å; 2.67 Å) 1 non-classical hydrogen bond (3.08 Å) 1 metal–acceptor bond (2.21 Å)
HApTi–calcium alendronate	7 classical hydrogen bonds (1.94 Å; 1.99 Å; 2.02 Å; 2.08 Å; 2.57 Å; 2.85 Å; 2.94 Å) 2 metal–acceptor bonds (3.0 Å; 3.22 Å)
HApTi–CaFb	6 classical hydrogen bonds (1.75 Å; 1.85 Å; 1.92 Å; 2.09 Å; 2.34 Å; 3.36 Å) 4 metal–acceptor bonds (2.04 Å; 2.89 Å; 3.16 Å; 3.36 Å) 3 acceptor–donor interactions (1.88 Å; 2.58 Å; 2.67 Å)

**Table 5:** Amount and percentage of calcium fructoborate and alendronate salts adsorbed on HAp and HApTi implants.

Implant Type	Adsorbed Substance	Implant Mass Before Surface Adsorption (g)	Implant Mass After Surface Adsorption (g)	Amount of Salt Adsorbed (g)	Percentage of Salt Adsorbed (%)
HAp	CaFb	0.3974	0.4214	0.0240	6.0393
	Sodium alendronate	0.3988	0.4199	0.0211	5.2909
	Calcium alendronate	0.3946	0.4112	0.0166	4.2068
HApTi	CaFb	0.3958	0.4320	0.0362	9.1460
	Sodium alendronate	0.3982	0.4276	0.0294	7.3832
	Calcium alendronate	0.3949	0.4187	0.0238	6.0268

## Data Availability

The original contributions presented in this study are included in the article. Further inquiries can be directed to the corresponding author.
